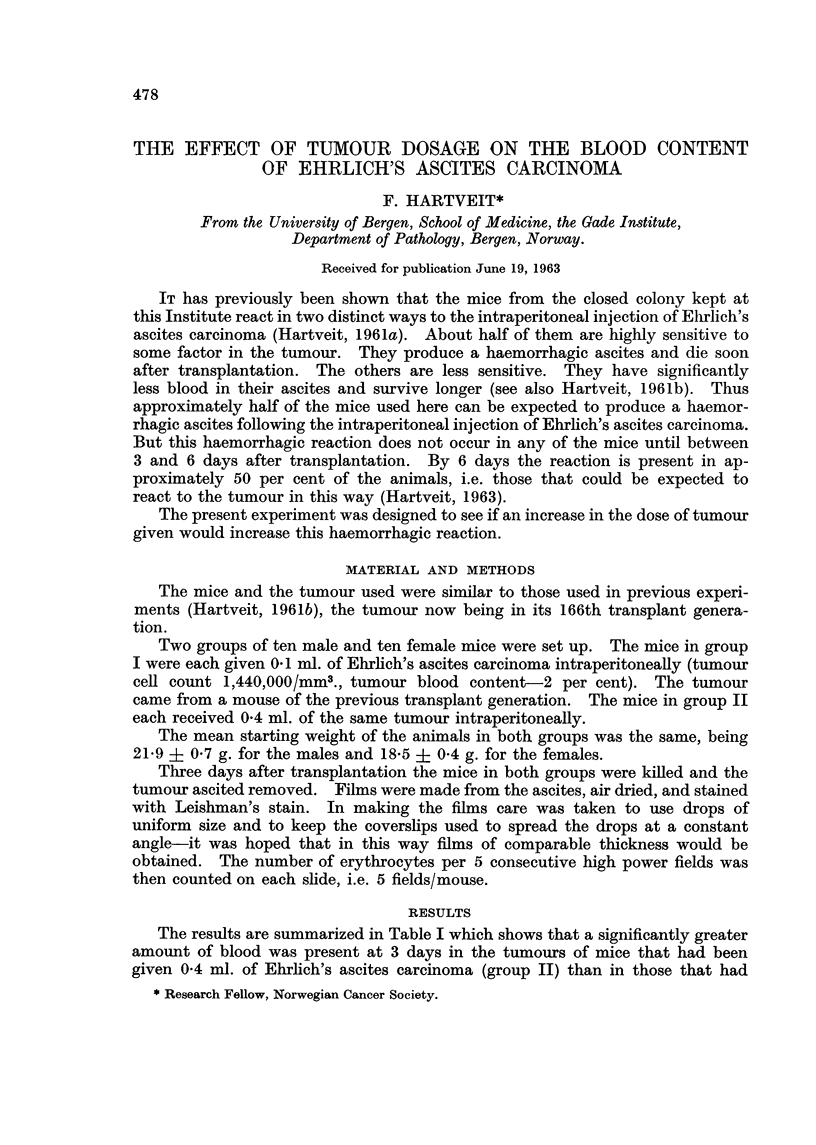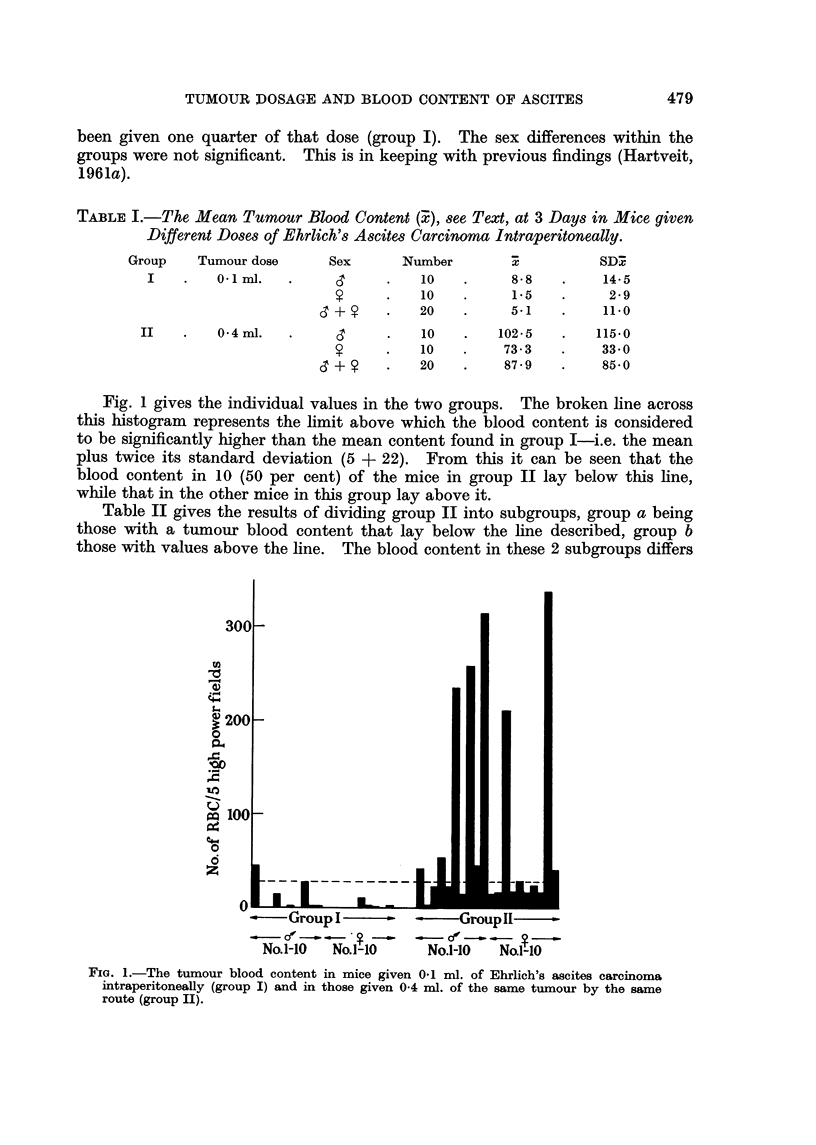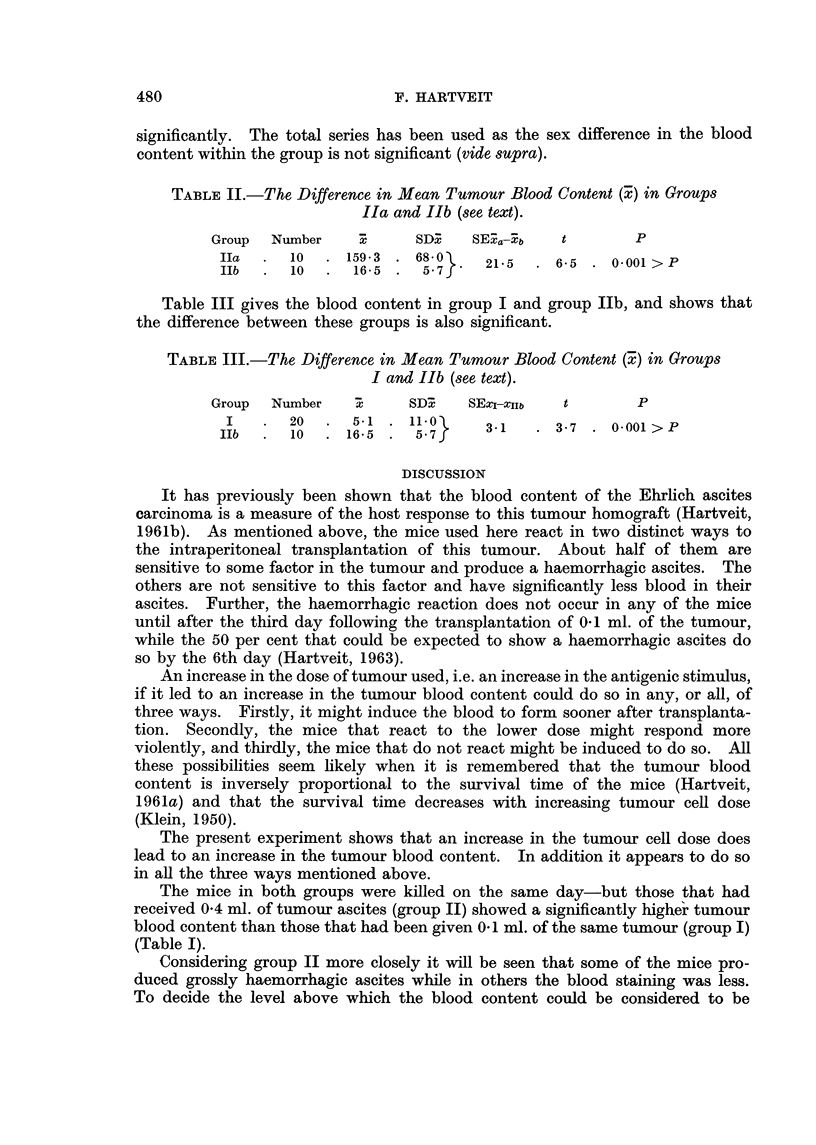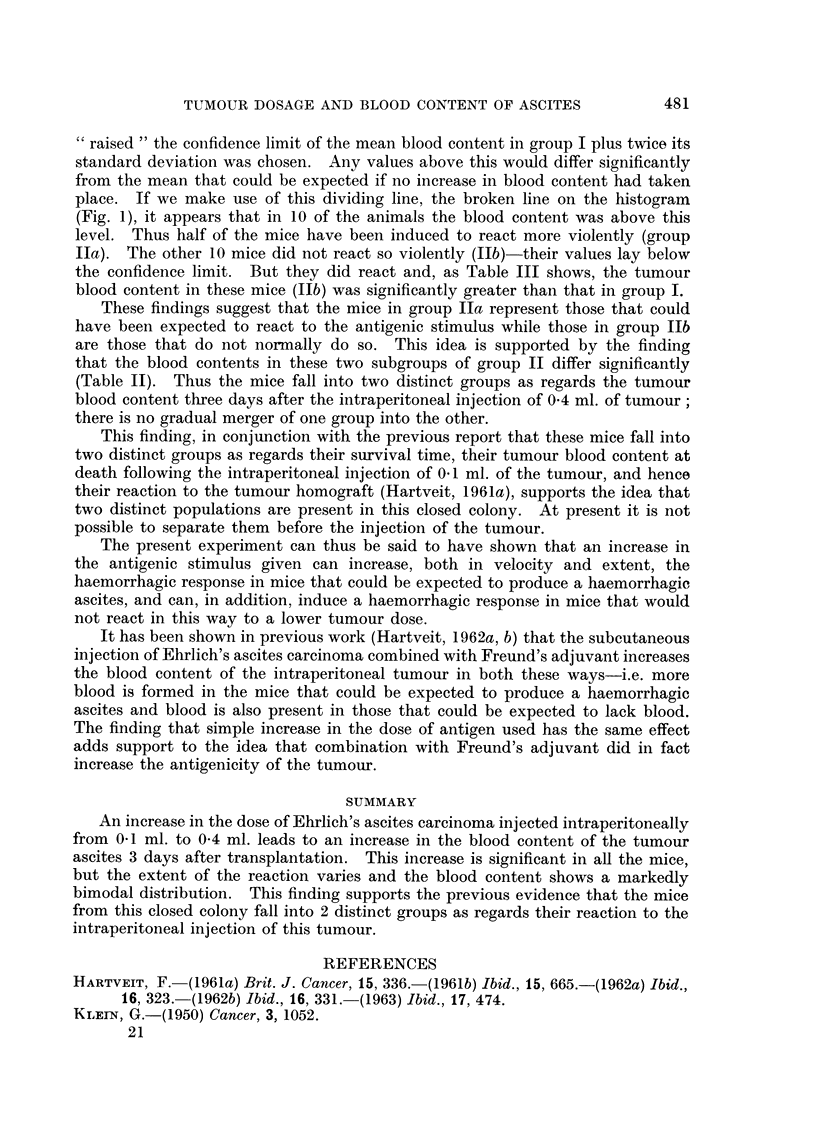# The Effect of Tumour Dosage on the Blood Content of Ehrlich's Ascites Carcinoma

**DOI:** 10.1038/bjc.1963.64

**Published:** 1963-09

**Authors:** F. Hartveit


### THE EFFECT OF TUMOUR DOSAGE ON THE BLOOD CONTENT OF EHRLICH'S ASCITES CARCINOMA

F. HARTVEIT*

*From the University of Bergen, School of Medicine, the Gade Institute, Department of Pathology, Bergen, Norway.*

Received for publication June 19, 1963

IT has previously been shown that the mice from the closed colony kept at this Institute react in two distinct ways to the intraperitoneal injection of Ehrlich's ascites carcinoma (Hartveit, 1961*a*). About half of them are highly sensitive to some factor in the tumour. They produce a haemorrhagic ascites and die soon after transplantation. The others are less sensitive. They have significantly less blood in their ascites and survive longer (see also Hartveit, 1961b). Thus approximately half of the mice used here can be expected to produce a haemorrhagic ascites following the intraperitoneal injection of Ehrlich's ascites carcinoma. But this haemorrhagic reaction does not occur in any of the mice until between 3 and 6 days after transplantation. By 6 days the reaction is present in approximately 50 per cent of the animals, i.e. those that could be expected to react to the tumour in this way (Hartveit, 1963).

The present experiment was designed to see if an increase in the dose of tumour given would increase this haemorrhagic reaction.

### MATERIAL AND METHODS

The mice and the tumour used were similar to those used in previous experiments (Hartveit, 1961*b*), the tumour now being in its 166th transplant generation.

Two groups of ten male and ten female mice were set up. The mice in group I were each given 0·1 ml. of Ehrlich's ascites carcinoma intraperitoneally (tumour cell count 1,440,000/mm$^3$., tumour blood content—2 per cent). The tumour came from a mouse of the previous transplant generation. The mice in group II each received 0·4 ml. of the same tumour intraperitoneally.

The mean starting weight of the animals in both groups was the same, being 21·9 $\pm$ 0·7 g. for the males and 18·5 $\pm$ 0·4 g. for the females.

Three days after transplantation the mice in both groups were killed and the tumour ascited removed. Films were made from the ascites, air dried, and stained with Leishman's stain. In making the films care was taken to use drops of uniform size and to keep the coverslips used to spread the drops at a constant angle—it was hoped that in this way films of comparable thickness would be obtained. The number of erythrocytes per 5 consecutive high power fields was then counted on each slide, i.e. 5 fields/mouse.

### RESULTS

The results are summarized in Table I which shows that a significantly greater amount of blood was present at 3 days in the tumours of mice that had been given 0·4 ml. of Ehrlich's ascites carcinoma (group II) than in those that had

* Research Fellow, Norwegian Cancer Society.

been given one quarter of that dose (group I). The sex differences within the groups were not significant. This is in keeping with previous findings (Hartveit, 1961*a*).

TABLE I.—*The Mean Tumour Blood Content* ($\bar{x}$), *see Text, at* 3 *Days in Mice given Different Doses of Ehrlich's Ascites Carcinoma Intraperitoneally.*

| Group | Tumour dose | Sex | Number | $\bar{x}$ | SD$\bar{x}$ |
|---|---|---|---|---|---|
| I | 0·1 ml. | ♂ | 10 | 8·8 | 14·5 |
| | | ♀ | 10 | 1·5 | 2·9 |
| | | ♂ + ♀ | 20 | 5·1 | 11·0 |
| II | 0·4 ml. | ♂ | 10 | 102·5 | 115·0 |
| | | ♀ | 10 | 73·3 | 33·0 |
| | | ♂ + ♀ | 20 | 87·9 | 85·0 |

Fig. 1 gives the individual values in the two groups. The broken line across this histogram represents the limit above which the blood content is considered to be significantly higher than the mean content found in group I—i.e. the mean plus twice its standard deviation (5 + 22). From this it can be seen that the blood content in 10 (50 per cent) of the mice in group II lay below this line, while that in the other mice in this group lay above it.

Table II gives the results of dividing group II into subgroups, group *a* being those with a tumour blood content that lay below the line described, group *b* those with values above the line. The blood content in these 2 subgroups differs

FIG. 1.—The tumour blood content in mice given 0·1 ml. of Ehrlich's ascites carcinoma intraperitoneally (group I) and in those given 0·4 ml. of the same tumour by the same route (group II).

significantly. The total series has been used as the sex difference in the blood content within the group is not significant (*vide supra*).

TABLE II.—*The Difference in Mean Tumour Blood Content* ($\bar{x}$) *in Groups IIa and IIb* (*see text*).

| Group | Number | $\bar{x}$ | $SD\bar{x}$ | $SE\bar{x}_a - \bar{x}_b$ | $t$ | $P$ |
|---|---|---|---|---|---|---|
| II*a* | 10 | 159·3 | 68·0 | 21·5 | 6·5 | $0 \cdot 001 > P$ |
| II*b* | 10 | 16·5 | 5·7 | | | |

Table III gives the blood content in group I and group IIb, and shows that the difference between these groups is also significant.

TABLE III.—*The Difference in Mean Tumour Blood Content* ($\bar{x}$) *in Groups I and IIb* (*see text*).

| Group | Number | $\bar{x}$ | $SD\bar{x}$ | $SEx_{I} - x_{IIb}$ | $t$ | $P$ |
|---|---|---|---|---|---|---|
| I | 20 | 5·1 | 11·0 | 3·1 | 3·7 | $0 \cdot 001 > P$ |
| II*b* | 10 | 16·5 | 5·7 | | | |

DISCUSSION

It has previously been shown that the blood content of the Ehrlich ascites carcinoma is a measure of the host response to this tumour homograft (Hartveit, 1961b). As mentioned above, the mice used here react in two distinct ways to the intraperitoneal transplantation of this tumour. About half of them are sensitive to some factor in the tumour and produce a haemorrhagic ascites. The others are not sensitive to this factor and have significantly less blood in their ascites. Further, the haemorrhagic reaction does not occur in any of the mice until after the third day following the transplantation of 0·1 ml. of the tumour, while the 50 per cent that could be expected to show a haemorrhagic ascites do so by the 6th day (Hartveit, 1963).

An increase in the dose of tumour used, i.e. an increase in the antigenic stimulus, if it led to an increase in the tumour blood content could do so in any, or all, of three ways. Firstly, it might induce the blood to form sooner after transplantation. Secondly, the mice that react to the lower dose might respond more violently, and thirdly, the mice that do not react might be induced to do so. All these possibilities seem likely when it is remembered that the tumour blood content is inversely proportional to the survival time of the mice (Hartveit, 1961*a*) and that the survival time decreases with increasing tumour cell dose (Klein, 1950).

The present experiment shows that an increase in the tumour cell dose does lead to an increase in the tumour blood content. In addition it appears to do so in all the three ways mentioned above.

The mice in both groups were killed on the same day—but those that had received 0·4 ml. of tumour ascites (group II) showed a significantly higher tumour blood content than those that had been given 0·1 ml. of the same tumour (group I) (Table I).

Considering group II more closely it will be seen that some of the mice produced grossly haemorrhagic ascites while in others the blood staining was less. To decide the level above which the blood content could be considered to be

" raised " the confidence limit of the mean blood content in group I plus twice its standard deviation was chosen. Any values above this would differ significantly from the mean that could be expected if no increase in blood content had taken place. If we make use of this dividing line, the broken line on the histogram (Fig. 1), it appears that in 10 of the animals the blood content was above this level. Thus half of the mice have been induced to react more violently (group II*a*). The other 10 mice did not react so violently (II*b*)—their values lay below the confidence limit. But they did react and, as Table III shows, the tumour blood content in these mice (II*b*) was significantly greater than that in group I.

These findings suggest that the mice in group II*a* represent those that could have been expected to react to the antigenic stimulus while those in group II*b* are those that do not normally do so. This idea is supported by the finding that the blood contents in these two subgroups of group II differ significantly (Table II). Thus the mice fall into two distinct groups as regards the tumour blood content three days after the intraperitoneal injection of 0·4 ml. of tumour ; there is no gradual merger of one group into the other.

This finding, in conjunction with the previous report that these mice fall into two distinct groups as regards their survival time, their tumour blood content at death following the intraperitoneal injection of 0·1 ml. of the tumour, and hence their reaction to the tumour homograft (Hartveit, 1961*a*), supports the idea that two distinct populations are present in this closed colony. At present it is not possible to separate them before the injection of the tumour.

The present experiment can thus be said to have shown that an increase in the antigenic stimulus given can increase, both in velocity and extent, the haemorrhagic response in mice that could be expected to produce a haemorrhagic ascites, and can, in addition, induce a haemorrhagic response in mice that would not react in this way to a lower tumour dose.

It has been shown in previous work (Hartveit, 1962*a*, *b*) that the subcutaneous injection of Ehrlich's ascites carcinoma combined with Freund's adjuvant increases the blood content of the intraperitoneal tumour in both these ways—i.e. more blood is formed in the mice that could be expected to produce a haemorrhagic ascites and blood is also present in those that could be expected to lack blood. The finding that simple increase in the dose of antigen used has the same effect adds support to the idea that combination with Freund's adjuvant did in fact increase the antigenicity of the tumour.

### SUMMARY

An increase in the dose of Ehrlich's ascites carcinoma injected intraperitoneally from 0·1 ml. to 0·4 ml. leads to an increase in the blood content of the tumour ascites 3 days after transplantation. This increase is significant in all the mice, but the extent of the reaction varies and the blood content shows a markedly bimodal distribution. This finding supports the previous evidence that the mice from this closed colony fall into 2 distinct groups as regards their reaction to the intraperitoneal injection of this tumour.

### REFERENCES

HARTVEIT, F.—(1961*a*) *Brit. J. Cancer*, **15**, 336.—(1961*b*) *Ibid.*, **15**, 665.—(1962*a*) *Ibid.*, **16**, 323.—(1962*b*) *Ibid.*, **16**, 331.—(1963) *Ibid.*, **17**, 474.

KLEIN, G.—(1950) *Cancer*, **3**, 1052.